# Effects of genotype and fattening system on the quality of male lamb meat – Part 1: Technological properties and carcass measurements

**DOI:** 10.5194/aab-62-605-2019

**Published:** 2019-12-20

**Authors:** Mehmet Sarı, Yüksel Aksoy, Kadir Önk, Hakan Erinç, Serpil A. Işık, Muammer Tilki

**Affiliations:** 1 Department of Animal Science, Faculty of Agriculture, Kırşehir Ahi Evran University, 40100, Kırşehir, Turkey; 2 Department of Animal Science, Faculty of Agriculture, Eskişehir Osmangazi University, 26160, Eskişehir, Turkey; 3 Department of Animal Science, Faculty of Veterinary Medicine, Kafkas University, 36000, Kars, Turkey; 4 Department of Food Engineering, Faculty of Engineering, Niğde Ömer Halisdemir University, 51240, Niğde, Turkey; 5 Department of Veterinary, Macka Vocational School, Karadeniz Technical University, 61750, Trabzon, Turkey

## Abstract

This study was conducted to determine the effect of genotype and fattening system on carcass measurements of lambs and technological properties of the male lamb meat (*Musculus longissimus dorsi*, MLD). The animal material in the study included 39 Hemşin (H) and 39 Tuj (T) male lambs. Extensive (E), semi-intensive (SI) and intensive (I) fattening systems were applied in the study, which was completed within 90 d. In the E, SI and I fattening groups, a total of 48 lambs, including 16 lambs in each group, were slaughtered. The results of the study indicated that the effect of genotype on the first-hour yellowness ($b^{*}$), being one of the colour parameters of the MLD, and the effect of the fattening system on 1 h hour redness ($a^{*}$) and chroma ($C^{*}$), being among the colour parameters, were statistically significant (P<0.05). The effect of genotype and fattening system on MLD pH at 45 min (pH${}_{45\;\min}$) and 24 h (pH${}_{24\;h}$) after the slaughtering and on the third and seventh hour drip loss (DL %) was statistically nonsignificant (P>0.05). The effect of genotype and fattening system on DL, cooking loss (CL %) and texture (TT) was nonsignificant (P>0.05), whereas the effect of these factors on water-holding capacity (WHC %) was significant (P<0.05). The effect of genotype on external carcass length (ECL), internal carcass length (ICL), internal hindquarter length (IHL), and carcass and leg conformation was statistically significant (P<0.05). The effect of the fattening system on all the carcass measurements except for carcass conformation, carcass depth (CD) and external chest width (ECW) was statistically significant (P<0.05). Genotype and fattening system affected the colour and some quality traits of meat and carcass measurements of lambs.

## 1 Introduction

Sheep breeding is an important source of income for low-income farmers in rural areas. Sheep are grazing animals which get most of their nutrient requirements from pasture. They are more economical than other animal species, are resistant to diseases and harsh environmental conditions, are easy to herd, and can effectively benefit from plant resources and vegetation which cannot be utilised by cattle in the region where they are reared. Sheep convert the plant resources into meat and milk, which are important for human nutrition, and they meet the needs of humans as a food of animal origin (Akcapinar, 2000; Gursoy, 2006).

Sheep breeding is one of the main sectors in the field of husbandry in which Turkey can compete with EU countries (Ozkan, 2006). As of 2018, the number of sheep in Turkey is reported to be 35 194 972 (TurkStat, 2019). With respect to sheep population, Turkey has a great potential in Europe thanks to the number of sheep. This, therefore, is an important potential opportunity for Turkey. However, sheep breeding in Turkey is done using traditional raising methods on indigenous breeds, having low productivity. In the traditional production systems, sheep rearing largely takes place in meadows and pastures of low quality where animals' nutritional requirements are met inadequately. This production model generally results in low productivity and in turn means that breeders make inadequate revenue. When considering Turkey's rapidly rising population, the need to consume more animal products also increases day by day. Within this context, increasing productivity in sheep breeding in Turkey and, for this purpose, carrying out intensive and semi-intensive breeding systems with optimum care and feeding conditions are required (Ekiz et al., 2012a; Sari et al., 2014).

In order for human beings to eat healthily, it is essential to take animal- and vegetable-based foods into the body at an adequate and balanced level. For this reason, it is very valuable to place great importance on meat production and quality (Unece Standard, 2006). There are many factors that affect the meat quality of lambs and therefore the value of the product. These factors are determined in two ways, including intrinsic factors, which are directly dependent on the animals, and extrinsic factors which the animals are exposed to during the breeding and pre-slaughter periods. While the intrinsic factors include breed, sex, slaughter weight, birth type, birth time, maternal age and genetical interventions, the extrinsic factors include the exercise situation of animals, feeding style, fattening system, and applications before and during slaughter (Sanudo et al., 1998; Akcapinar and Ozbeyaz, 1999; Hoffman et al., 2003; Carrasco et al., 2009; Esenbuga et al., 2009; Aksoy et al., 2019).

The consumption habits of societies change as their level of education and living standards increase. Socio-economic development leads to people giving more importance to quality. The quality level of meat and carcass differs between breeders and consumers. Breeders place importance on carcass weight, conformation and fattiness level, whereas consumers prioritise properties of meat such as the colour of meat at purchase, the cooking loss during cooking, and the texture and the aroma of meat during eating (Murray, 1995; Vergara and Gallego, 1999).

The technological properties of lamb meat might be affected by genotype and fattening system (Santos-Silva et al., 2002; Karaca et al., 2016; Ekiz et al., 2019). So, genotype and fattening system have great importance in obtaining quality lamb meat. Although studies have been made to assess slaughter and carcass traits of Hemşin and Tuj lambs (Sari et al., 2012, 2015; Onk et al., 2017), there is no evidence for traits of technological properties of meat ($L^{*}$ (lightness), $a^{*}$ (redness) and $b^{*}$ (yellowness); pH; cooking loss; drip loss; and water holding capacity). This study was conducted to determine the effect of genotype and fattening system on the technological properties of meat and carcass measurements of male lambs.

## 2 Materials and methods

The study was conducted at the Research and Application Farm Sheep Breeding Unit at Kafkas University's Faculty of Veterinary Science Education. Table 1 shows the number of lambs (fattened and slaughtered) in each group and subgroup formed based on genotype and fattening system. The animal material in the study included 78 male lambs, including 39 male lambs (extensive (E): 13; semi-intensive (SI): 13; and intensive (I): 13) from the Hemşin genotype and 39 male lambs (E: 13; SI: 13; and I: 13) from the Tuj (T) genotype. Lambs were cared for in accordance with rules established by the Local Animal Studies Ethics Committee of Kafkas University in Kars, Turkey. Each subgroup was composed of the same number of lambs, and there were no losses of lambs during fattening. H lambs were supplied by a breeder in Artvin province. The Hemşin and Tuj breeds, being the indigenous breeds, are well-adapted to conditions where feed is scarce. The Hemşin sheep have long fat tails at the base and have the longest tail among the indigenous breeds. The Tuj breed, on the other hand, is a fat-tailed sheep breed. Both breeds are raised throughout north-eastern Turkey generally for their meat. T lambs were supplied by Kafkas University's Faculty of Veterinary Science Education Research and Application Farm. Before the study, all of the lambs were immunised for internal and external parasites. The fattening process began 10 d after they had adjusted to their pasture and feed.

**Table 1:** The number of lambs (fattened and slaughtered) in each group and subgroup of genotypes and fattening systems.

Parameters	Fattened	Slaughtered	Initial	Slaughter
	groups (n)	groups (n)	weight (kg)	weight (kg)
G				kg
T	39	24	20.85	37.51
H	39	24	22.77	38.52
FS				
E	26	16	21.64	31.92
SI	26	16	21.86	41.47
I	26	16	21.94	40.67
G and FS				
T and E	13	8	20.86	31.13
T and SI	13	8	20.73	41.55
T and I	13	8	20.95	39.85
H and E	13	8	22.42	32.71
H and SI	13	8	22.99	41.38
H and I	13	8	22.92	41.49
Total	78	48	21.81	38.01

T: Tuj; H: Hemşin; G: genotype; FS: fattening system.

In the E and SI fattening groups, the lambs were grazed on pasture for 8 h d${}^{-1}$. The lambs in the SI fattening group were fed ad libitum with both pastures and concentrated feed. The concentrated feed was given to each lamb in the I fattening group ad libitum along with 270.00 g of grass hay per day. The concentrated feed contained 17.10 % crude protein (CP) and 2900 kcal kg${}^{-1}$ metabolic energy (ME) (NRC, 1985). Tables 2 and 3 show the content and the nutritional content of the concentrated feed and roughage. While the concentrated feed was prepared in a private feed factory, roughage was provided from the Veterinary Science Faculty farm. The feeds were analysed in the Veterinary Science Faculty, Department of Animal Feeding and Nutrition Diseases, at Kafkas University. The lambs in the I fattening group were continuously given clean water; those in E and SI groups were given clean water at least three times a day.

**Table 2:** The composition of concentrated feed used in semi-intensive and intensive fattening groups.

Ingredient	kg t${}^{-1}$	Crude	Metabolic
		protein (%)	energy
			(kcal kg${}^{-1}$)
Barley	320	12	3110
Maize bran	100	9.2	2740
Maize	180	10	3300
Vegetable oil	26	–	8500
Sunflower cake	60	37	2250
Cotton seed cake	60	34	2300
Soy cake	140	48	3310
Molasses	85	7.8	2580
Limestone	20	–	–
Sodium bicarbonate	2	–	–
Salt	5	–	–
Vit.–min. premix	2	–	–

**Table 3:** The nutritional contents of concentrate feed and roughage (%).

Ingredient	Concentrated feed	Roughage
DM (%)	88.80	90.69
CP (%)	17.10	10.35
CC (%)	5.7	32.38
CF (%)	3.5	2.00
CA (%)	6.4	8.86
ME (kcal kg${}^{-1}$)${}^{*}$	2910	2000

DM: dry matter; CP: crude protein; CC: crude cellulose; CF: crude fat; CA: crude ash; ME: metabolic energy. ${}^{*}$ It was calculated based on the table values.

Table 4 shows the natural nutritional content of the pasture upon which the animals were grazed at different mowing times. For this purpose, the samples were taken from four parts of the pasture three times, once a month (between 5 June and 5 August). For this, 50 cm${}^{2}$ grass of the pasture was cut using a grass cutter at 1 cm above the ground level. The dry matter (DM), crude protein (CP), crude ash (CA), crude cellulose (CC), crude fat (CF) and nitrogen-free extract (NFE) levels were determined according to AOAC (1990).

**Table 4:** The natural nutritional content of the pasture, on which the lambs grazed, for different mowing times (%).

	DM	OM	CA	CP	CF	CS	NFE
Mow I	26.25	23.85	2.30	3.55	0.69	8.40	11.35
Mow II	32.35	30.10	2.30	2.70	0.99	9.70	16.68
Mow III	36.40	33.90	2.75	3.50	1.05	12.66	16.70

DM: dry matter; OM: organic matter; CP: crude ash; CP: crude protein; CF: crude fat; CC: crude cellulose; NFE: nitrogen-free extract.

It took 90 d to complete the fattening process. A total of 48 male lambs including 24 from H genotype (E: 8; SI: 8; and I: 8) and 24 from T genotype (E: 8; SI: 8; and I: 8) were then slaughtered at Kafkas University's Faculty of Veterinary Science slaughterhouse and their meat quality and carcass measurements were examined.

After the slaughtering process, external carcass length (ECL), external hindquarter length (EHL), external chest width (ECW), chest depth (CD), hindquarter width (HW), hindquarter depth (HD) and hindquarter perimeter (HP) were measured from the external carcass surface, whereas internal carcass length (ICL), internal chest width (ICW) and internal hindquarter length (IHL) from the internal carcass surface were measured (Russo et al., 2003; Vacca et al., 2008; Carrasco et al., 2009; Yakan and Unal, 2010a). The carcass measurements were obtained using a measuring cane and tape. In the study, carcass conformation (internal chest width, ICW; internal carcass length, ICL) and leg conformation (hindquarter width, HW; internal hindquarter length, IHL) were determined in accordance with Pena et al. (2005), Vacca et al. (2008) and Santos et al. (2013).

After the slaughtering process, 45 min (pH${}_{45\;\min}$) and 24 h (pH${}_{24\;h}$) pH values from the *M. longissimus dorsi* (MLD) were measured, from a section opened in three points on the muscle between the 12th and 13th ribs in the right half of carcass (Testo 205, Germany; Hoffman et al., 2003; Ramirez and Cava, 2007).

Additionally, colour parameters ($L^{*}$ (lightness), $a^{*}$ (redness) and $b^{*}$ (yellowness)) of the *M. longissimus et thoracis* (at the level of the 12th and 13th ribs) were determined at 1 h and 24 h (Konica Minolta CR-400, Minolta, Japan) after slaughtering (Sen et al., 2011). The chroma was measured based on the same muscle parameters (chroma = $C^{*}(a^{*2}+b^{*2}{)}^{1/2}$), whereas the hue (hue angle $H^{*}$ = ${\mathrm{tan}}^{-1}(b^{*}/a^{*})$) values were calculated according to Aksoy and Ulutas (2016).

In the study, the water holding capacity (WHC) of the MLD was determined using the press method. For this purpose, fatty tissue and connective tissue were cleaned and ground; a 2–3 g MLD meat sample was weighed and then put between two sheets of filter paper (Whatman Grade 1 Qualitative). Glass plates were placed above and below the sheets of filter paper, and then a weight of 2.250 kg was placed on it for 5 min in order to determine WHC (%) (Barton-Gade et al., 1993).

The MLD samples (about 30 g) were vacuumed within vacuum bags and stored at +4 ${}^{\circ }$C for 3 and 7 d. At the end of the target periods, the drip loss % (DL) was determined (Aksoy and Ulutas, 2016).

Pieces (50 g) were cut from the MLD, weighed and then vacuumed into vacuum bags. Afterwards, the bags were boiled in a water bath of +80 ${}^{\circ }$C until the internal temperature of the meat was determined to have reached +70 ${}^{\circ }$C using a digital thermometer. Once the samples were cooked, they were kept under running tap water for about 30 min until they cooled down to ambient temperature (+25 ${}^{\circ }$C), and then the cooking loss (CL) percent was measured (Honikel, 1998; Mitchaothai et al., 2006; Lu et al., 2019). Following this process, the meat hardness (TT) value of cubic (1 cm${}^{3}$) boiled meat samples, cut and prepared in parallel to the muscle fibres, was determined using a TA.XP*plusC* Texture Analyser (Stable Micro Systems, Godalming, UK) (Martinez et al., 2004).

In the study, the least squares method was used in SPSS 12.0 packaged software in order to determine the effects of genotype (H and T) and fattening system (E, SI and I) on the carcass measurements of lambs and technological properties of the MLD (SPSS Inc., 2003). One-way analysis of variance and Duncan's test were used to determine whether or not there was a significant difference among the groups.

In the least squares method, the following model was used:

1 $$Y_{ijk}=\mu +a_{i}+b_{j}+ab{(}_{ij})+e_{ijk},$$

where $Y_{ijk}$ represents the yield value of any animal, μ is the expected average value, $a_{i}$ is the effect of genotype (i: H and T), $b_{j}$ is the effect of fattening system (j: E, SI and I), $ab{(}_{ij})$ is the effect of the genotype–fattening system interaction and $e_{ijk}$ represents the error margin based on chance.

## 3 Results

Table 5 shows $L^{*}$, $a^{*}$, $b^{*}$, $C^{*}$ and $H^{*}$ changes related to MLD colour parameters. Measurements of the MLD samples made at 1 h and 24 h indicated that there was no statistically significant difference between the genotype groups, except for $b^{*}$  at 1 h (P<0.05). Also, no statistically significant difference was found among the measurements for the different fattening systems, except for 1 h $a^{*}$  and $C^{*}$ colour measurements (P<0.05). The effect of the genotype–fattening system interaction on colour parameters was statistically nonsignificant (P>0.05).

**Table 5:** The means and standard errors of least squares related to the M. *longissimus dorsi* (MLD) colour parameters of the Tuj (T) and Hemşin (H) lambs in different fattening systems.

Traits	n	1 h					24 h				
		($L^{*}$)	($a^{*}$)	($b^{*}$)	($C^{*}$)	($H^{*}$)	($L^{*}$)	($a^{*}$)	($b^{*}$)	($C^{*}$)	($H^{*}$)
G	P value	ns	ns	${}^{*}$	ns	ns	ns	ns	ns	ns	ns
T	24	33.87±0.24	11.91±0.25	1.79±0.12	12.05±0.26	8.45±0.47	38.48±0.31	13.21±0.17	3.44±0.14	13.66±0.18	14.55±0.50
H	24	34.04±0.23	11.32±0.20	1.45±0.11	11.42±0.21	7.18±0.46	38.43±0.47	13.46±0.14	3.37±0.16	13.89±0.13	14.07±0.66
FS	P value	ns	${}^{*}$	ns	${}^{*}$	ns	ns	ns	ns	ns	ns
E	16	33.79±0.25	$12.07\pm 0.23^{a}$	1.74±0.12	$12.20\pm 0.25^{a}$	8.14±0.46	38.02±0.42	13.10±0.16	3.56±0.13	13.58±0.14	15.24±0.60
SI	16	33.99±0.34	$11.05\pm 0.32^{b}$	1.46±0.12	$11.15\pm 0.32^{b}$	7.40±0.49	38.79±0.51	13.37±0.23	3.27±0.20	13.78±0.24	13.68±0.75
I	16	34.08±0.27	$11.72\pm 0.26^{\mathrm{ab}}$	1.66±0.19	$11.86\pm 0.28^{\mathrm{ab}}$	7.90±0.79	38.55±0.53	13.53±0.16	3.38±0.20	13.97±0.18	14.01±0.77
G and FS	P value	ns	ns	ns	ns	ns	ns	ns	ns	ns	ns
T and E	8	34.05±0.35	12.17±0.34	1.81±0.18	12.31±0.35	8.39±0.71	38.54±0.52	13.16±0.14	3.65±0.18	13.67±0.13	15.50±0.77
T and SI	8	33.73±0.47	11.29±0.53	1.59±0.17	11.41±0.55	7.96±0.72	38.12±0.42	13.04±0.39	3.05±0.28	13.40±0.42	13.06±1.02
T and I	8	33.82±0.45	12.25±0.37	1.97±0.26	12.43±0.39	9.00±1.06	38.78±0.68	13.44±0.29	3.64±0.21	13.93±0.33	15.08±0.61
H and E	8	33.53±0.35	11.97±0.33	1.67±0.17	12.09±0.35	7.89±0.63	37.51±0.64	13.04±0.29	3.47±0.19	13.50±0.26	14.97±0.95
H and SI	8	34.25±0.48	10.81±0.35	1.31±0.15	10.90±0.36	6.85±0.63	39.46±0.89	13.71±0.17	3.50±0.29	14.16±0.19	14.30±1.13
H and I	8	34.34±0.30	11.19±0.28	1.35±0.24	11.29±0.30	6.80±1.09	38.31±0.83	13.63±0.17	3.13±0.33	14.02±0.16	12.93±1.36

${}^{a,\;b,\;c}$ The difference between groups in the same column with different letters is significant (P<0.05). ns: P>0.05; ${}^{*}$ P<0.05. T: Tuj; H: Hemşin; G: genotype; FS: fattening system. $L^{*}$: lightness; $a^{*}$: redness; $b^{*}$: yellowness. E: extensive; SI: semi-intensive; I: intensive.

Table 6 shows the mean and the standard errors of pH, DL, WHC and TT parameters of the meat. When the pH${}_{45\;\min}$ and pH${}_{24\;h}$ values of the MLD were examined, it was observed that the effect of genotype and fattening system was not statistically significant (P>0.05). Also, the third and seventh day DL% effect of genotype and fattening system was statistically nonsignificant (P>0.05). It was determined that only the effect of genotype and fattening system on WHC was statistically significant (P<0.05). The effect of the genotype–fattening system interaction on pH, DL, WHC, CL and TT values was statistically nonsignificant (P>0.05).

**Table 6:** The effect of the different fattening systems on some of the meat quality properties of the M. *longissimus dorsi* (MLD) of the Tuj (T) and Hemşin (H) lambs.

Traits	n	pH		WHC (%)	CL (%)	DL (%)		n	TT
		45 min	24 h			third day	seventh day		(kg cm${}^{-2}$)
G	P value	ns	ns	${}^{**}$	ns	ns	ns		ns
T	24	6.40±0.05	5.88±0.02	31.35±0.45	29.06±0.72	9.96±0.98	11.14±0.38	22	8.18±0.41
H	24	6.47±0.04	5.68±0.21	33.13±0.54	29.67±0.94	9.04±0.49	11.33±0.44	23	8.17±0.41
FS	P value	ns	ns	${}^{*}$	ns	ns	ns		ns
E	16	6.42±0.05	5.93±0.03	$33.64\pm 0.52^{a}$	28.88±0.86	8.04±0.57	11.24±0.49	15	8.45±0.50
SI	16	6.52±0.04	5.86±0.04	$31.66\pm 0.68^{b}$	30.29±1.06	10.39±1.40	11.50±0.48	14	7.85±0.52
I	16	6.36±0.07	5.54±0.25	$31.43\pm 0.61^{b}$	28.92±1.14	10.07±0.55	10.99±0.54	16	8.23±0.48
G and FS	P value	ns	ns	ns	ns	ns	ns		ns
T and E	8	6.32±0.06	5.91±0.03	32.20±0.64	30.30±0.49	8.77±0.94	11.40±0.65	7	8.30±0.73
T and SI	8	6.54±0.08	5.87±0.05	30.31±0.76	29.43±0.99	11.14±2.79	11.49±0.80	7	8.41±0.73
T and I	8	6.36±0.11	5.85±0.04	31.55±0.87	27.46±1.81	9.98±0.57	10.54±0.50	8	7.84±0.68
H and E	8	6.52±0.05	5.95±0.05	35.08±0.36	27.46±1.54	7.31±0.59	11.08±0.76	8	8.61±0.68
H and SI	8	6.51±0.10	5.85±0.07	33.00±0.95	31.15±1.90	9.64±0.62	11.51±0.57	7	7.28±0.73
H and I	8	6.37±0.09	5.22±0.63	31.31±0.91	30.39±1.30	10.15±0.99	11.42±0.98	8	8.62±0.68

${}^{a,\;b,\;c}$ The difference between groups in the same column with different letters is significant (P<0.05). ns: P>0.05; ${}^{*}$ P<0.05; ${}^{**}$ P<0.01. T: Tuj; H: Hemşin; G: genotype; FS: fattening system. WHC: water holding capacity; CL: cooking loss; DL: drip loss; TT: texture. E: extensive; SI: semi-intensive. I: intensive.

Table 7 shows the effect of genotype and the fattening system on some carcass measurements and carcass conformation. The effect of genotype on ECL, ICL, IHL, carcass conformation and leg conformation was statistically significant (P<0.05). It was determined that the effect of the fattening system on ECL, ICL, EHL, IHL, HP, ICW, HW, HD and leg conformation was statistically significant (P<0.05). The effect of the genotype–fattening system interaction on other carcass measurements except for HP and CD was statistically nonsignificant (P>0.05).

**Table 7:** The effect of the fattening systems on the carcass measurements (cm) and conformation coefficients of the Tuj (T) and Hemşin (H) lambs.

Carcass Measurements	G		FS			P value		
	T	H	E	SI	I	G	FS	G and FS
	(n=24)	(n=24)	(n=16)	(n=16)	(n=16)			
ECL	56.85±0.63	62.01±0.39	$57.42\pm 0.97^{b}$	$60.96\pm 0.74^{a}$	$59.90\pm 0.81^{a}$	${}^{***}$	${}^{***}$	ns
ICL	62.02±0.53	64.03±0.50	$62.61\pm 0.56^{b}$	$64.59\pm 0.74^{a}$	$61.88\pm 0.55^{b}$	${}^{**}$	${}^{**}$	ns
EHL	42.40±0.25	41.69±0.35	$41.71\pm 0.25^{b}$	$42.89\pm 0.39^{a}$	$41.54\pm 0.41^{b}$	ns	${}^{*}$	ns
IHL	38.97±0.25	39.79±0.26	$39.04\pm 0.29^{b}$	$40.33\pm 0.23^{a}$	$38.77\pm 0.33^{b}$	${}^{*}$	${}^{***}$	ns
HP	60.62±0.73	60.66±0.46	$57.98\pm 0.59^{b}$	$62.19\pm 0.45^{a}$	$61.74\pm 0.69^{a}$	ns	${}^{***}$	${}^{**}$
CD	24.32±0.25	24.81±0.20	24.29±0.30	24.87±0.23	24.53±0.32	ns	ns	${}^{*}$
ECW	20.71±0.34	19.97±0.35	20.28±0.30	20.03±0.55	20.72±0.42	ns	ns	ns
ICW	28.93±0.26	28.74±0.24	$28.39\pm 0.29^{b}$	$29.54\pm 0.30^{a}$	$28.58\pm 0.27^{b}$	ns	${}^{*}$	ns
HW	21.08±0.24	21.02±0.14	$20.23\pm 0.18^{b}$	$21.42\pm 0.20^{a}$	$21.49\pm 0.20^{a}$	ns	${}^{***}$	ns
HD	10.66±0.21	10.90±0.27	$9.92\pm 0.20^{b}$	$11.31\pm 0.21^{a}$	$11.11\pm 0.34^{a}$	ns	${}^{***}$	ns
Carcass Conformation								
ICW and ICL for carcass conformation	0.47±0.003	0.45±0.003	0.45±0.004	0.46±0.004	0.46±0.004	${}^{***}$	ns	ns
HW and IHL for leg conformation	0.54±0.006	0.53±0.004	$0.52\pm 0.003^{b}$	$0.53\pm 0.005^{b}$	$0.56\pm 0.006^{a}$	${}^{*}$	${}^{***}$	ns

${}^{a,\;b,\;c}$ The difference between groups in the same line with different letters is significant (P<0.05). ns: P>0.05; ${}^{*}$ P<0.05; ${}^{**}$ P<0.01; ${}^{***}$ P<0.001. T: Tuj; H: Hemşin; G: genotype; FS: fattening system. E: extensive; SI: semi-intensive; I: intensive. ECL: external carcass length; ICL: internal carcass length; EHL: external hindquarter length; IHL: internal hindquarter length; HP: hindquarter perimeter; CD: chest depth; ECW: external chest width; ICW: internal chest width; HW: hindquarter width; HD: hindquarter depth.

## 4 Discussion

Meat colour is one of the characteristics that consumers take into consideration in the purchasing stage (Mancini and Hunt, 2005). Dark-coloured meats are described as aged animal meats by consumers; because of this description and the hardness of the meat, these characteristics may affect consumption preferences. In this study, it was determined that the effect of genotype on all the colour characteristics except for the yellow colour coordinate $b^{*}$ at 1 h was nonsignificant (P>0.05). The meat of T lambs had a more yellow colour compared to H lambs in at 1  h. The effect of genotype on $L^{*}$, $a^{*}$, $C^{*}$ and $H^{*}$ values for the MLD at 1 h after the slaughtering was nonsignificant. Esenbuga et al. (2009) reported similar results for Awassi and Morkaraman lambs. On the other hand, Abdullah et al. (2011) reported that the effect of genotype and crossbreeding on $L^{*}$, $a^{*}$, $b^{*}$ and $H^{*}$ values was significant. In this study, it was determined that the effect of the fattening system on all the colour characteristics except for $a^{*}$  at 1 h and $C^{*}$  at 1 h was nonsignificant. At 1 h after slaughtering, $a^{*}$ and $C^{*}$ values for the meat were higher in lambs in the E fattening group compared to the SI and I groups. The $L^{*}$, $a^{*}$, $b^{*}$, $C^{*}$ and $H^{*}$ values of all three fattening systems determined in the present study were lower than the same values determined by Aguayo-Ulloa et al. (2013) in the commercial and traditional fattening systems. This difference may be associated with differences in breed, fattening period, slaughter weight, care and feeding.

The final pH (pH${}_{24\;h}$) value of meat is reported to be one of the most important meat quality characteristics due to its effects on meat colour, hardness value, WHC, CL and DL. For this reason, it is desirable that pH${}_{24\;h}$ should be between 5.50 and 5.80 to prevent defective meat formation (PSE (pale–soft–exudative) and DFD (dark–firm–dry)) (Aksoy and Ulutas, 2016; Yakan and Unal, 2010b). In the study, pH${}_{45\;\min}$ values of the MLD varied between 6.40 and 6.47, and its pH${}_{24\;h}$ value varied between 5.68 and 5.88 in both genotype groups. The pH values determined for T and H lambs in the present study were similar to the values determined by Caneque et al. (2004) for the Manchega breed. Similarly to the present study, Esenbuga et al. (2009) and Celik and Yilmaz (2010) reported that the effect of breed on pH values was statistically nonsignificant. In contrast, Aksoy et al. (2019) determined that the effect of breed on pH${}_{24\;h}$ was statistically significant. The study revealed that the difference between the fattening systems in terms of pH variance was nonsignificant. Similarly, Diaz et al. (2002) and Priolo et al. (2002) reported that the differences between the fattening systems, in terms of MLD pH values, based on pasture and concentrated feed were statistically nonsignificant. Aguayo-Ulloa et al. (2013) stated that the differences between commercial and traditional fattening systems in terms of pH${}_{24\;h}$ were statistically nonsignificant. However, Ekiz et al. (2012b) determined that the differences between the breeding systems in terms of pH${}_{24\;h}$ values of Kivircik lambs were significant.

In this study, the effect of genotype and the fattening system on third and seventh day DL was found to be nonsignificant. Similarly, Esenbuga et al. (2009) and Celik and Yilmaz (2010) reported that the effect of breed on DL was not significant, and Rodriguez et al. (2008) and Ekiz et al. (2012b) reported that the effect of different breeding systems on DL was not significant. The present study revealed that the effect of genotype and fattening system on WHC determined in the MLD at 24 h after slaughtering was significant. WHC for the MLD was higher in T lambs compared to H lambs and higher in lambs in the SI and I fattening groups compared to those lambs in the E fattening group. In other words, the meat of H genotype sheep released more water than T genotype sheep, and the meat of lambs in the E fattening group released more water than those in the SI and I fattening groups. In terms of WHC, Tuj lambs (in genotype) and the SI and I fattening groups should be preferred. Similarly, Santos-Silva et al. (2002) reported that the different fattening systems had an effect on WHC, and the WHC of the MLD in lambs in the E fattening group was higher compared to those in the SI and I fattening groups. However, Diaz et al. (2002) reported that different fattening systems had no effect on WHC. Also, Velasco et al. (2004) stated that the difference between the lambs fed with pasture + barley and pasture + concentrate feed in terms of WHC was not statistically significant. In this study, it was determined that the effect of genotype and fattening system on CL was nonsignificant. The CL value determined in the H and T genotypes in this study was higher compared to the CL value of the MLD determined by Yarali et al. (2014) for Kivircik lambs. CL values determined in both genotypes in the present study were lower than the CL value reported by Esenbuga et al. (2001) for T lambs slaughtered at the weight of 30 kg and similar to the value reported by them for the Awassi breed. CL determined in the E and I fattening groups in the present study was lower than the value reported by Soycan Onenc et al. (2015) for the Sakiz breed in the same fattening group. The CL results determined in the present study were different than other studies, and this was associated with the differences in breed, slaughter weight, slaughter age, content of rations, cooking temperature of the meat and cooking time. Texture is one of the factors affecting the edibility of meat. In the study, it was determined that the effect of genotype and fattening system on T lambs was nonsignificant. The meat having the TT value higher than 5.5 kg cm${}^{-2}$ is considered to be tough (Aksoy and Ulutas, 2016). In this study, TT values of H and T genotypes were determined to be 8.17 and 8.18 kg cm${}^{-2}$. This pointed out that the meat of both genotypes was a bit tough. These values were higher than the values reported by Esenbuga et al. (2001) for Awassi, Morkaraman and Awassi × Tuj breeds and similar to the values reported by them for the T genotype. TT values obtained in each of three fattening systems in the present study were higher than the values reported by Santos-Silva et al. (2002) for Merino Branco and Île de France × Merino Branco breeds in the pasture, pasture + concentrate feed and concentrate feed groups. TT results determined in the present study were different from the other studies due to the differences in breed, slaughter weight, slaughter age, content of rations, cooking temperature of meat, cooking time, model of the cutting tool, cutting speed and stroke height of the cutting tool.

In the study, it was determined that the effect of genotype on ECL and ICL was significant. The ECL and ICL values for H lambs were higher than those for T lambs. This revealed that the H genotype had a longer body form. Additionally, it was found that the effect of fattening systems on all other measurements except for CD, ECW and carcass conformation was significant. ECL, ICL and EHL values in the E fattening group determined in the present study were higher compared to the carcass length I (49.70, 49.60 and 49.10), carcass length II (58.10, 57.80 and 57.60) and leg length (38.90, 39.30 and 38.50 cm) values reported by Ulusan and Aksoy (1996) for Morkaraman, Morkaraman × Tuj and Tuj male yearling lambs grazing on pasture. The EHL value of the I fattening group determined in the present study was similar to the value (41.00 cm) reported by Altinel et al. (1998) for Kivircik lambs in the same fattening system. On the other hand, the IHL value for the I fattening group determined in the present study was higher than the value (25.20 cm) reported by Altinel et al. (1998) for the same fattening system. Also, EHL, IHL and HW values for the I fattening group in this study were higher than EHL (37.33 and 39.66), IHL (28.00 and 28.00 cm) and HW (18.16 and 18.33 cm) values reported by Esen and Yildiz (2000) for Akkaraman and Sakiz × Akkaraman crossbreed (F${}_{1}$) lambs in the I fattening group. EHL and HW values from the I fattening group determined in the present study were lower than EHL (47.66, 49.10 and 48.40 cm) and HW (26.04, 24.90 and 24.40 cm) values reported by Ozbey and Akcan (2003) for Morkaraman, Sakiz × Morkaraman and Kivircik × Morkaraman lambs in the I fattening group. Additionally, IHL and HP values determined in this study were higher than IHL (32.30, 32.30 and 31.10 cm) and HP (28.64, 30.06 and 28.68 cm) reported by the same researchers.

The ECL value of the E fattening group determined in this study was higher than the value reported by Alvarez et al. (2013) for the same fattening system; on the other hand, the ECW value was similar to those determined by the same researchers. The leg and carcass conformation values for the E and I fattening groups determined in the present study were lower than the leg and carcass conformation values reported by Borton et al. (2005) for the E and I fattening groups. ICL, HP, ECW, HW, carcass compactness and carcass conformation values determined in each of three fattening systems in this study were higher than those reported by Carrasco et al. (2009) for Churra Tensina lambs in different fattening systems, whereas the leg conformation was lower than the value determined by the same researchers. The values of the technological properties of MLD and carcass measurements of male lambs were different than the other studies due to differences in breed, fattening systems, fattening period, care, feeding and slaughter weight.

In this study, carcass measurements of male lambs and the technological properties of their meat were determined for the first time by applying different fattening systems for H and T lambs. It was observed that genotype and fattening system did not affect pH, DL, CL or TT values of the meat. However, the WHC of the meat, which is used as an indicator of taste, microbial quality and production potential (Abdullah and Al-Najdawi, 2005), was higher in T lambs compared to H lambs and was higher in the lambs in the SI and I fattening groups compared to those in the E fattening group. In addition, although there was no statistical difference in the study, CL value was higher in H lambs than T lambs and higher in the lambs in the SI fattening group compared to those in the E and I fattening groups. In the study, it was observed that while H lambs had higher ECL, ICL and IHL values, T lambs had higher carcass and leg conformation. In general, lambs in the SI and I fattening groups had higher values in carcass measurements compared to the lambs in the E fattening group. It was found that genotype and different fattening systems affected some technological properties of the meat, and the technological properties of the meat were usually similar to the values in meat from lambs from other indigenous breeds reared in Turkey and those in the limited number of different fattening systems. Consequently, considering the consumer preferences, H lambs in the semi-intensive system can be preferred because of the high $L^{*}$ colour value and low hardness value of meat.

## Data Availability

The data sets are available upon request from the corresponding author.
